# Global Prevalence of Domestic Cat Hepadnavirus: An Emerging Threat to Cats’ Health?

**DOI:** 10.3389/fmicb.2022.938154

**Published:** 2022-06-24

**Authors:** Maya Shofa, Yasuyuki Kaneko, Kazuki Takahashi, Tamaki Okabayashi, Akatsuki Saito

**Affiliations:** ^1^Laboratory of Veterinary Microbiology, Department of Veterinary Science, Faculty of Agriculture, University of Miyazaki, Miyazaki, Japan; ^2^Graduate School of Medicine and Veterinary Medicine, University of Miyazaki, Miyazaki, Japan; ^3^Faculty of Agriculture, Veterinary Teaching Hospital, University of Miyazaki, Miyazaki, Japan; ^4^Center for Animal Disease Control, University of Miyazaki, Miyazaki, Japan

**Keywords:** domestic cat hepadnavirus, novel hepadnavirus, prevalence, FeLV, FIV

## Abstract

Hepatitis B is an infectious hepatocellular disease of global concern caused by hepatitis B virus (HBV), which belongs to *Hepadnaviridae*. Recently, a novel HBV-like virus, domestic cat hepadnavirus (DCH), was detected from an immunocompromised cat with a hepatic disease in Australia. Subsequent molecular investigation by independent research groups revealed that its prevalence rates were 6.5% in Australia, 10.8% in Italy, 12.4% in Thailand, 12.3% in Malaysia, 3.08% in the United Kingdom, and 0.78% in Japan. Although the correlation between DCH infection and hepatic diseases remains to be elucidated, understanding the diversity of circulating DCH will contribute to its prevention and control in domestic cats. Herein, we summarize the current epidemiological data of DCH in these countries.

## Introduction

*Hepadnaviridae* has partially double-stranded, circular DNA as genome and infects several animal species, including mammals, reptiles, frogs, birds, and fish (Magnius et al., 2020). In humans, chronic hepatitis B virus (HBV) infection, which is a major public health concern, can induce liver damage, including cirrhosis and hepatocellular carcinoma (HCC; Liang, 2009). In 2018, an HBV-like virus was identified in a domestic cat with large cell lymphoma in Sidney, Australia. This novel member of *Hepadnaviridae*, domestic cat hepadnavirus (DCH), belongs to the *Orthohepadnavirus* genus (Aghazadeh et al., 2018).

**Figure 1:**
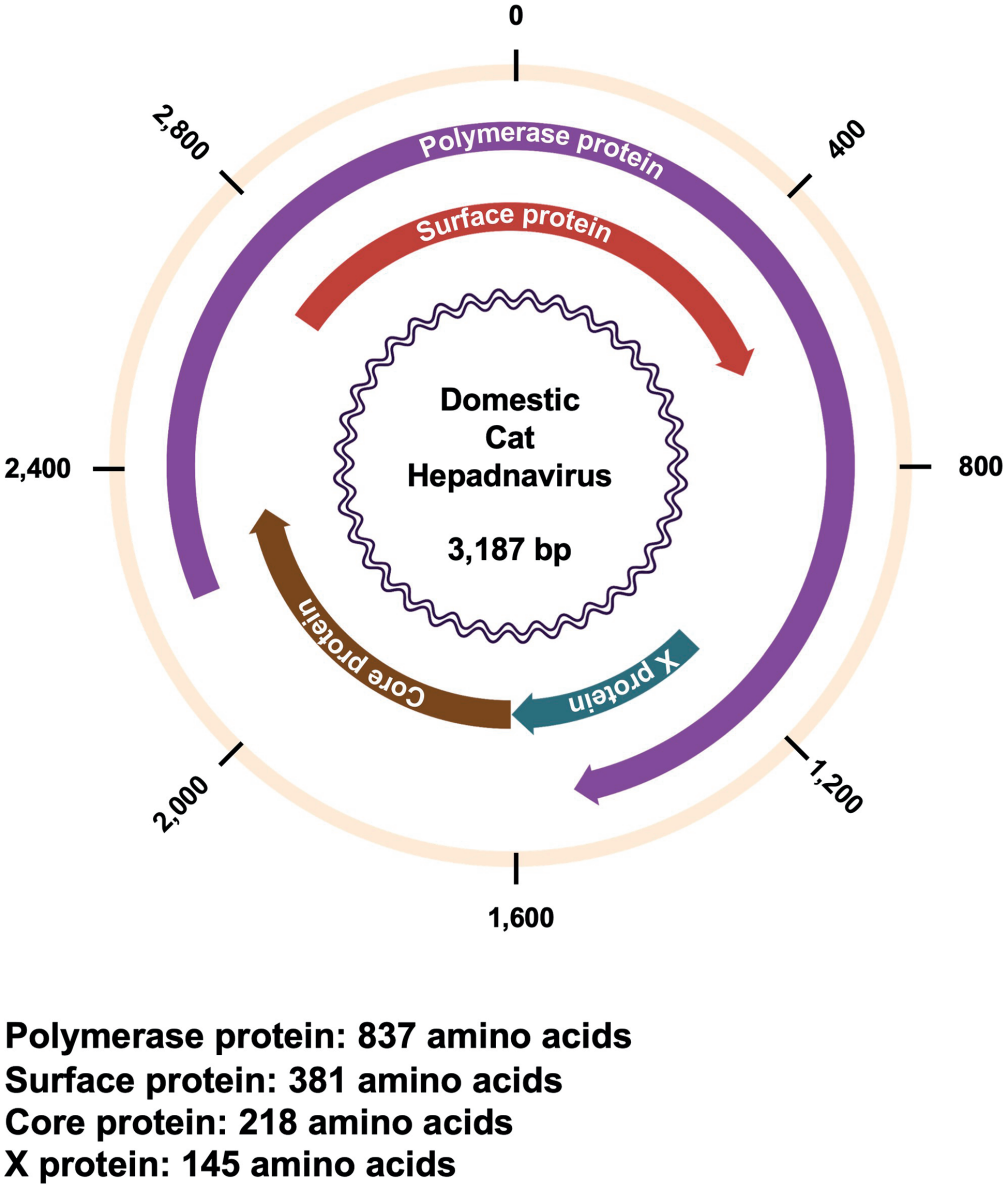
Genomic structure of DCH. Genomic structure of DCH showing the position and orientation of each viral protein. The length of each viral protein is shown below.

The genome of DCH is ~3,200 bases in length, with partially double-stranded, circular DNA (Aghazadeh et al., 2018; Figure 1). Similar to other Orthohepadnaviruses, the DCH genome encodes four overlapping open reading frames for polymerase (P), surface (S), core (C), and X proteins (Aghazadeh et al., 2018; Magnius et al., 2020). In HBV, P proteins are multifunctional enzymes with a protein-priming function, both RNA- and DNA-dependent DNA synthesis function, and ribonuclease H activity (Clark et al., 2021). S proteins (or HBsAg) play an important role in viral attachment and host immune response manipulation (Churin et al., 2015). Based on their different domains and glycosylation status, S proteins are divided into large (LHBs), medium (MHBs), and small (SHBs) proteins (Churin et al., 2015). Although C proteins are relatively small proteins (183 residues in HBV) that mainly function in viral capsid assemblage, they also have several functions in viral life cycle and may modulate the epigenetics of hosts (Zlotnick et al., 2015). Moreover, X proteins are multifunctional regulatory proteins that modulate protein degradation pathways, apoptosis, transcription, signal transduction, cell cycle progress, and genetic stability (van Hemert et al., 2011; Zhang et al., 2019). Although DCH and HBV are grouped together in genus *Orthohepadnavirus*, the function of each viral protein of DCH remains to be elucidated since there is a large difference between DCH and HBV (Aghazadeh et al., 2018; Ko et al., 2020; Piewbang et al., 2020).

Since its first identification, surveys on DCH prevalence in domestic cats have been conducted in several countries. Herein, we summarize the current knowledge on the global prevalence and tropism of DCH to understand the current prevalence and epidemiology of DCH infection.

## Global Prevalence of DCH

**Figure 2:**
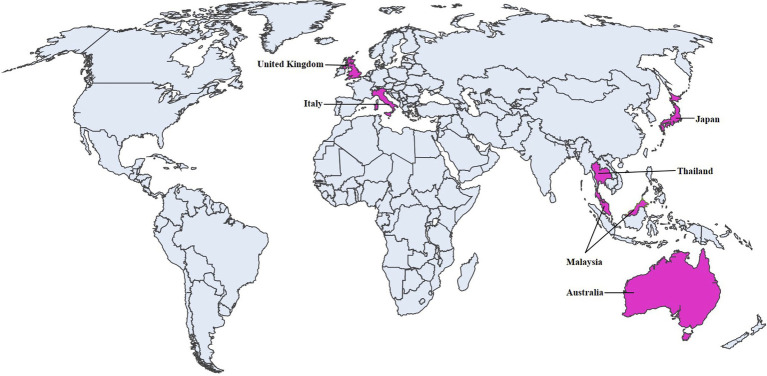
Geographical distribution of DCH. Prevalence of DCH. Countries with DCH-positive case are shown in pink.

Since DCH was first identified in 2018 in Australia (Aghazadeh et al., 2018), subsequent surveys were conducted to determine its prevalence in several countries. The surveys revealed positive cases in Australia, Italy, Thailand, Malaysia, United Kingdom, and Japan (Figure 2). The current geographic distributions of DCH are summarized in Table 1, and the current findings in each country were described.

**Table 1:** Current geographical distribution and prevalence of DCH.

No	Country	Year reported	Prevalence	Surveillance	Clinical significance	Associated co-infection	References
1	Australia	2018	6.5%	Hospital based investigation	lymphoma	FIV	Aghazadeh et al., 2018
2	Italy	2019	10.8%	University based investigation	viremia	FeLV or FIV	Lanave et al., 2019
		2022	4.2	University based investigation	Elevated ALT	–	Scavone et al., 2022
		2022	9.8% 25%^*^	University based investigation	DCH Seropositive	–	Fruci et al., 2022
3	Thailand	2020	12.4%	Hospital based investigation	viremia	FeLV or FIV	Piewbang et al., 2020
4	Malaysia	2021	14.9%	University based investigation	Elevated ALT	FeLV	Anpuanandam et al., 2021
5	United Kingdom	2021	3.08%	Hospital based investigation	Uveitis	–	Jeanes et al., 2022
6	Japan	2022	0.78%	University based investigation	Elevated ALT	–	Takahashi et al., 2022

* Seroprevalence.

### Australia

DCH was first identified in 2018 in Sydney in a transcriptomic study of a castrated, seven-year-old, male cat on a virus discovery project. This cat, which had symptoms of vomiting and weight loss, was diagnosed with large B-cell lymphoma. Notably, this cat was infected with feline immunodeficiency virus (FIV). Subsequently, molecular screening of DCH was conducted by an independent research group. The group demonstrated that the positivity rate of DHC in FIV positive cats was 10% (6/60), while in FIV negative cats was 3.2% (2/63; Aghazadeh et al., 2018).

### Italy

DCH was identified in 10.8% (42/390) of cat serum samples in 2019 (Lanave et al., 2019). Molecular screening identified a higher DCH positivity (17.8%) in sera submitted for diagnostic tests for infectious diseases than with sera submitted for pre-surgical tests (5.1%). Furthermore, 45.2% (14/31) of DCH-positive cats were also positive for FIV and/or feline leukemia virus (FeLV), suggesting a correlation between retroviral infection and DCH infection (Lanave et al., 2019).

Following the initial study, a longitudinal observation of two DCH-positive cats for 4.5 months or 11 months, respectively, demonstrated that DCH infections can be chronic, which is similar to HBV infections in humans (Capozza et al., 2021). Moreover, a persistent alanine aminotransferase (ALT) elevation was also documented, which is an indication of liver diseases. However, retrospective investigation showed that only a small proportion of cats with liver diseases was positive for DCH. Furthermore, DCH was not detected in cats with a high likelihood of liver diseases (Scavone et al., 2022). These observations imply that DCH infection has a limited contribution on hepatic diseases in cats. Recent serological surveillance using recombinant DCH core antigens detected 25% seropositive cats from a total of 256 cat serum samples (Fruci et al., 2022), suggesting that DCH infection can be an inapparent infection.

Interestingly, a novel hepadnavirus was also detected from dogs in 2022; it was tentatively named domestic dog hepadnavirus (DDH). The genome of DDH shared 98.0 and 96.9% nucleotide (nt) identity at the full genome level with Italian and Australian DCH, respectively (Diakoudi et al., 2022). This survey revealed that 6.3% (40/635) of dog sera were DDH-positive (Diakoudi et al., 2022). Out of 40 DCH positive sera, only 23 samples have information related to hematological and serum biochemical assays which showed 47.8% (11/23) of DDH-positive dogs had an increased ALT (Diakoudi et al., 2022). This report proposes the possibility of inter-species transmission of hepadnavirus in carnivores.

### Malaysia

In Malaysia, a molecular survey demonstrated a prevalence of DCH in shelter and pet cats. Among 340 samples, DCH was confirmed in 12.3% (31/253) of blood samples and 14.9% (13/87) of liver samples (Anpuanandam et al., 2021). Moreover, the study demonstrated that breed and sex were not risk factors for DCH infection, while FeLV infection was significantly associated with DCH positivity in Malaysia. Similar to HBV infection in humans, nearly 50% of DCH-positive cats showed elevated ALT levels (Kim et al., 2008; Anpuanandam et al., 2021). DCH infection was also positively correlated with the age of cats, and its prevalence in pet cats was higher than in shelter cats (Anpuanandam et al., 2021). The reason for the difference in DCH prevalence between pet cats and shelter cats remains unclear at this moment.

### Thailand

In 2020, the occurrence of DCH in Thailand was investigated by PCR using 209 randomized domestic cat sera samples and 15 sera samples from cats with liver damage. Among the samples, 12.4% (26/209) of sera and 20% (3/15) sera and necropsied organs were confirmed to be positive for pan-hepadnavirus and DCH (Piewbang et al., 2020). Similar to findings in Malaysia (Anpuanandam et al., 2021), the prevalence of DCH in liver tissue samples was higher than that in blood samples. Moreover, Thai group also confirmed that DCH infection was associated with retroviral infection. Among DCH-positive samples, 57.7% were positive for FIV, 11.5% were positive for FeLV, and 3.8% were positive to both retroviruses (Piewbang et al., 2020). These results emphasize that retrovirus infections are positively correlated with DCH infection.

### United Kingdom

In 2019, a collaborative study was conducted to investigate the correlation of DCH infection with chronic hepatitis and HCC in cats. The multicenter investigation evaluated the presence of DCH in liver biopsy collections from diseased or healthy cats from four institutions of four countries, including the United States, United Kingdom, Australia, and New Zealand. Using PCR test, DCH was detected in 43% (6/14) of chronic hepatitis and 28% (8/29) of HCC cases, whereas all samples from cholangitis (*n* = 6), biliary carcinoma (*n* = 18), and normal liver (*n* = 15) were negative for DCH. With an *in situ* hybridization (ISH) technique, DCH was detected in 100% (8/8) of chronic hepatitis and 33% (2/6) of HCC cases, whereas all samples from cholangitis (*n* = 5), biliary carcinoma (*n* = 11), and normal liver (*n* = 3) were negative for DCH. These results demonstrated an association between DCH infection and pathological lesions, which were similar to those observed in HBV-associated disease (Pesavento et al., 2019). These results support the speculation that DCH causes chronic hepatitis and HCC in cats.

Furthermore, a study was conducted to evaluate the involvement of DCH infection in uveitis. Previous studies demonstrated that HBV and Hepatitis C virus co-infection increases the risk of uveitis (Tien et al., 2016). Although no statistically significant difference was observed, DCH infection was detected in 2 of 65 cats with endogenous uveitis, while it was undetected in healthy controls (Jeanes et al., 2022).

### Japan

A recent study based on PCR using 139 blood samples from indoor cats identified one DCH-positive sample (positive rate: 0.78%; Takahashi et al., 2022). The positive sample was from a 17-year-old female cat with acute neuropathy and persistent ALT elevation before death. This cat had no health problems and no previous history of overseas travel. Moreover, X protein sequence analysis demonstrated that the virus was genetically different from strains in other countries. This finding suggests that the DCH infection in this cat occurred in Japan. Unlike the previous reports from other countries, this DCH-positive case had no FIV and/or FeLV co-infection. Furthermore, a research group from The University of Tokyo in Japan identified a DCH infection in a cat with fever with unknown origin (Genbank accession no. LC685967.1; Unpublished data).

## Phylogenetic Analysis of DCH

Genomic analysis confirmed that DCH is classified to the *Orthohepadnavirus* genus. Sixteen available genome sequences derived from DCH-positive samples in Australia, Italy, Malaysia, Thailand, and Japan, showing 96.4%–99.7% nucleotide similarity with each other. Recently, a putative DCH recombinant strain was reported in Thailand, suggesting a possible role of recombination in DCH evolution (Piewbang et al., 2020).

**Figure 3:**
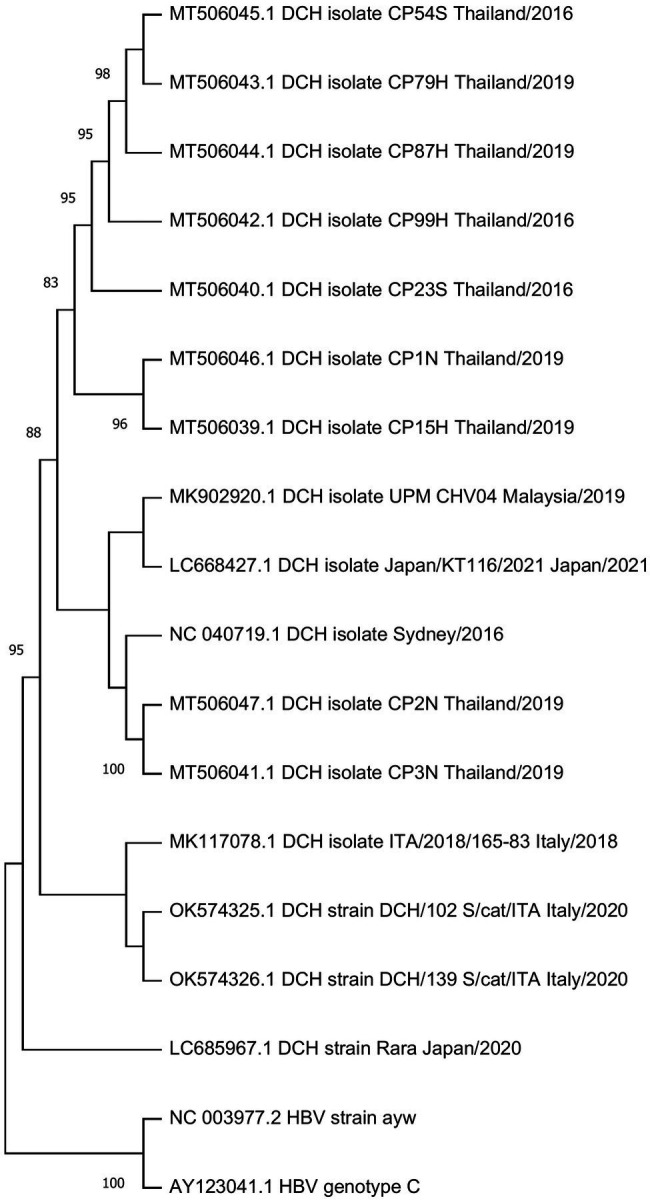
Phylogenetic tree of DCH. A phylogenetic tree was constructed using the complete sequences of available DCH sequences from Genbank. HBV strain ayw and HBV genotype C were included as outgroup. The tree was constructed using the maximum-likelihood method with a general time reversible nucleotide substitution model implemented in a MEGA X program (Kumar et al., 2018). Bootstrap values over 70% (1,000 replicates) are shown.

Interestingly, DCH strains in Thailand clustered into two different lineages. While one is close to the prototypic Sidney lineage, the other one generated its own lineage (Figure 3). The Malaysian strain was genetically close to the Japanese strain. Interestingly, while the Japanese strain DCH Japan/KT116/2021 grouped into the Australian lineage, another Japanese strain (Genbank accession no. LC685967.1) formed a novel lineage separate from all previous strains. Phylogenetic analysis revealed the genetic diversity of DCH genome according to the country of origin.

## Viral Tropism and Distribution of DCH in Organs

A retrospective investigation of 71 formalin-fixed, paraffin-embedded biopsies from cats with selected liver disease specimens showed a high detection rate of DCH in cats with chronic hepatitis (43%) and HCC (28%), whereas histologically normal biliary tract and liver-associated diseases were DCH-negative in PCR tests (Pesavento et al., 2019). In addition, ISH assay validated the presence of DCH in areas of liver inflammation and neoplasia (Pesavento et al., 2019).

Aside from the liver, DCH DNA was also detected in various organs (i.e., heart, lungs, intestines, kidneys, and spleen) by qPCR, and HBcAg expression was confirmed by immunohistochemistry (IHC; Piewbang et al., 2020). IHC staining also showed systemic inflammatory diseases in various organs, such as hepatitis, hepatic fibrosis, glomerulonephritis, lymphoplasmacytic enteritis, and histiocytic lymphadenitis (Piewbang et al., 2020). Furthermore, the intracellular localization of DCH particles was also observed by TEM analysis, suggesting that DCH may have a broad cell tropism. The association between DCH infection and liver diseases in cats needs further investigations.

## Route of DCH Infection and Transmission

As observed with HBV infection in humans, DCH infection has been correlated to FIV/FeLV infection, which leads to an immunocompromised condition (Aghazadeh et al., 2018; Lanave et al., 2019; Piewbang et al., 2020; Anpuanandam et al., 2021). Unlike HBV, which can be transmitted to other cats through blood, skin, and other bodily fluids, including serum, saliva, and semen through sexual or maternal route (Bancroft et al., 1977; Scott et al., 1980; Hou et al., 2005), DCH has only been detected in serum, whole blood, heart, lungs, intestines, kidneys, and spleen (Aghazadeh et al., 2018; Lanave et al., 2019; Piewbang et al., 2020; Anpuanandam et al., 2021). A longitudinal observation of two cats naturally infected with DCH showed that repeated PCR assays of oral, conjunctival, preputial, and rectal swabs were negative for DCH (Capozza et al., 2021). However, the fact that the DCH genome was detected in the blood suggests that it was potentially transmitted to other cats through blood. Despite the absence of DCH genome in rectal swabs (Capozza et al., 2021), a viral tropism study identified DCH immunoreactive signals in the cytoplasm of cryptal epithelia and endothelial cells within the lamina propria of intestinal villi (Piewbang et al., 2020). In addition, a qPCR assay also detected a high viral copy of DCH genome in intestinal samples (Piewbang et al., 2020). Further investigations on the possible route of DCH infection and transmission are needed to elucidate the risk factors of infection and prevent its transmission in family and breeding facilities. Moreover, the risk of DCH transmission should be seriously considered in the importation and exportation of cats.

## Conclusion

**Figure 4:**
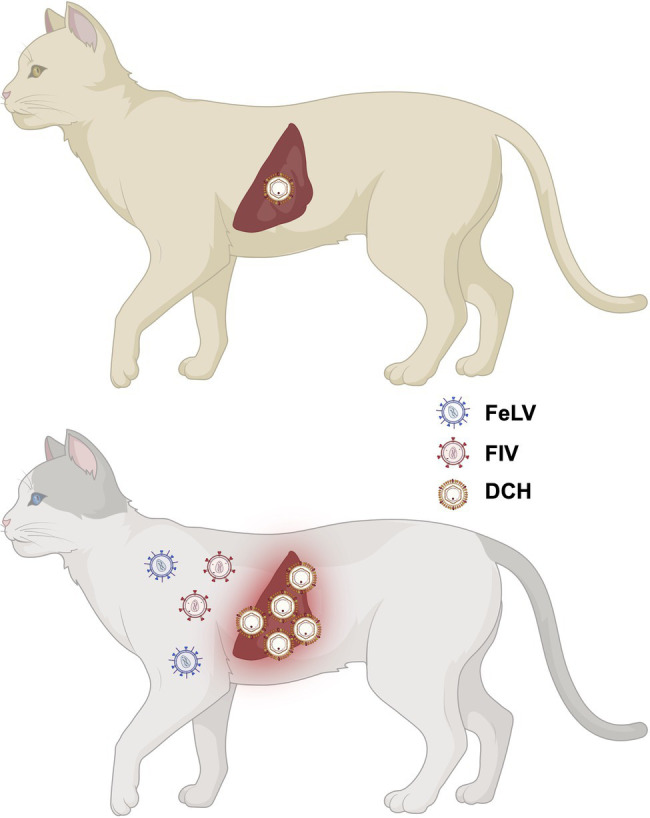
Possible impact of FIV/FeLV co-infection or pre-infection on DCH infection. Previous studies suggested that FIV/FeLV infection is associated with a higher chance of DHC infection.

Since its first identification in 2018 from an FIV-positive cat, DCH infection was identified in cats with and without hepatic diseases form other countries. Importantly, DCH viremia may pose an unexpected risk in transfusion or transplantation. Although DCH was identified in various organs of cats with chronic hepatitis, further studies on the viral tropism and pathobiology of DCH are still needed to clarify its pathogenicity. Further, accumulating evidence suggests that FIV/FeLV co-infection or pre-infection are risk factors for the acquisition of DCH infection (Figure 4). The effect of FIV/FeLV infection on DCH infection needs to be clarified. In addition, further epidemiological investigations in wider host species are required to understand the genetic variation and inter-species transmission events of DHC. The development of a rapid diagnosis kit may contribute to this investigation. Finally, further understanding of possible transmission routes is required. We believe that these investigations will facilitate the design of prevention strategies and contribute in maintaining the health of domestic cats.

## Author Contributions

MS: writing—original draft, investigation, editing, conceptualization, and data curation. YK, KT, and TO: editing and review. AS: conceptualization, editing, review, supervision, methodology, and investigation writing. All authors contributed to the article, read and approved the submitted version.

## Funding

This work was supported by grants from Japan Society for the Promotion of Science (JSPS) KAKENHI Grant-in-Aid for Scientific Research (C) 19K06382 (to AS); KAKENHI Grant-in-Aid for Scientific Research (B) 21H02361 (to TO and AS); KAKENHI Grant-in-Aid for Scientific Research (B) 22H02500 (to AS); and from a Grant for Joint Research Projects of the Research Institute for Microbial Diseases, Osaka University (to AS).

## Conflict of Interest

The authors declare that the research was conducted in the absence of any commercial or financial relationships that could be construed as a potential conflict of interest.

## Publisher’s Note

All claims expressed in this article are solely those of the authors and do not necessarily represent those of their affiliated organizations, or those of the publisher, the editors and the reviewers. Any product that may be evaluated in this article, or claim that may be made by its manufacturer, is not guaranteed or endorsed by the publisher.
